# Monocyte to high-density lipoprotein cholesterol ratio is associated with cerebral small vessel diseases

**DOI:** 10.1186/s12883-023-03524-9

**Published:** 2024-01-04

**Authors:** Ki-Woong Nam, Hyung-Min Kwon, Han-Yeong Jeong, Jin-Ho Park, Kyungha Min

**Affiliations:** 1 Department of Neurology, Seoul National University College of Medicine, 101 Daehak-Ro, Jongno-Gu, Seoul, 03080 South Korea; 2 Department of Neurology, Seoul Metropolitan Government-Seoul National University Boramae Medical Center, 20 Boramae-Ro 5-Gil, Dongjak-Gu, Seoul, 07061 South Korea; 3 Department of Family Medicine, Seoul National University College of Medicine and Seoul National University Hospital, 101 Daehak-Ro, Jongno-Gu, Seoul, 03080 South Korea

**Keywords:** Monocyte, Cholesterol, Inflammation, Endothelium, Atherosclerosis, Cerebral ischemia

## Abstract

**Background:**

Inflammation is a major pathological mechanism underlying cerebrovascular disease. Recently, a new inflammatory marker based on the ratio between monocyte count and high-density lipoprotein (HDL) cholesterol has been proposed. In this study, we evaluated the relationship between monocyte-to-HDL cholesterol ratio (MHR) and cerebral small vessel disease (cSVD) lesions in health check-up participants.

**Methods:**

This study was a retrospective cross-sectional study based on a registry that prospectively collected health check-up participants between 2006 and 2013. Three cSVD subtypes were measured on brain magnetic resonance imaging. White matter hyperintensity (WMH) volume, and lacunes and cerebral microbleeds (CMBs) were quantitatively and qualitatively measured, respectively. The MHR was calculated according to the following formula: MHR = monocyte counts (× 10^3^/μL) / HDL cholesterol (mmol/L).

**Results:**

In total, 3,144 participants were evaluated (mean age: 56 years, male sex: 53.9%). In multivariable analyzes adjusting for confounders, MHR was significantly associated with WMH volume [*β* = 0.099, 95% confidence interval (CI) = 0.025 to 0.174], lacune [adjusted odds ratio (aOR) = 1.43, 95% CI = 1.07–1.91], and CMB (aOR = 1.51, 95% CI = 1.03–2.19). In addition, MHR showed a positive quantitative relationship with cSVD burden across all three subtypes: WMH (*P* < 0.001), lacunes (*P* < 0.001), and CMBs (*P* < 0.001).

**Conclusions:**

High MHR was closely associated with cSVD in health check-up participants. Because these associations appear across all cSVD subtypes, inflammation appears to be a major pathological mechanism in the development of various cSVDs.

**Supplementary Information:**

The online version contains supplementary material available at 10.1186/s12883-023-03524-9.

## Background

Cerebral small vessel disease (cSVD) is a subclinical pathology commonly observed in current health examinations due to aging and the development of brain imaging technology [1]. cSVD increases the risk of dementia and stroke, and has received clinical attention [2, 3]. However, unlike those previously known to be asymptomatic, several studies have recently revealed that cSVD itself can cause cognitive impairment, dysphagia, and gait disturbance if the disease burden increases [4]. Therefore, studies have been conducted to identify common pathological mechanisms and risk factors that penetrate various cSVD subtypes [white matter hyperintensity (WMH), lacunes of presumed vascular origin, and cerebral microbleed (CMB)] [5–7].

Inflammation is one of these pathological mechanisms underlying the development of cSVD [8]. Chronic inflammation affects large and small cerebral vessels through various mechanisms including endothelial dysfunction, atherosclerosis, and thrombus formation [7, 9]. This phenomenon also occurs in the perforating artery that can cause cSVD; thus, various inflammatory markers are closely related to cSVD [10–12]. In particular, recently, novel inflammatory markers created by combining various inflammatory cells or biomarkers in consideration of the underlying pathological mechanism, such as the neutrophil-to-lymphocyte ratio (NLR), platelet-to-lymphocyte ratio, and systemic immune-inflammation index, have also been shown to be closely related to cSVD [13–15].

The monocyte-to-high-density lipoprotein (HDL) cholesterol ratio is also one of these novel markers [16]. Monocytes are cells that play an important role in chronic inflammation, and HDL cholesterol has properties that inhibit several functions of monocytes [17, 18]. Therefore, the ratio between them can more clearly reflect the inflammatory function of monocytes. Based on these properties, the monocyte-to-HDL cholesterol ratio (MHR) has been shown to be associated with various metabolic syndromes, atherosclerosis, and cardiovascular and cerebrovascular diseases in several studies [17–24]. However, studies analyzing the association between cSVD, subclinical cerebrovascular disease, and MHR are still lacking [25].

In this study, we evaluated the association between MHR values and radiological lesions of cSVD in health check-up participants. By examining the association between each subtype of cSVD and MHR, we examined whether MHR is a risk factor that applies to all cSVD pathologies or a risk factor that reflects only a specific pathology. We expected that these results would confirm the possibility of MHR in selecting high-risk groups requiring a diagnosis of cSVD. In addition, the potential of MHR as a biomarker for cSVD was evaluated compared with NLR, a well-known inflammatory biomarker.

## Methods

### Study population

Based on the prospectively collected health check-up registry data from the Seoul National University Hospital Health Promotion Center, we evaluated participants who underwent brain magnetic resonance imaging (MRI) between January 2006 and December 2013 (*n* = 3,257). As part of a health check-up, this center has been conducting extensive demographic, clinical, laboratory, and radiological evaluations [13]. Among them, the following participants were excluded based on the exclusion criteria: 1) having a history of stroke or severe neurological disease (*n* = 45), 2) age under 30 years (*n* = 7), 3) with missing data for major variables (*n* = 4), 4) history of severe systemic inflammatory conditions (e.g., hemato-oncologic disease, severe hepatic or renal disease, major surgery or trauma, use of immunosuppressant, and active infection within two weeks) (*n* = 57) [13]. Resultantly, 3,144 health check-up participants were included in the final analyses.

Korean medical services are characterized by high accessibility to brain imaging and relatively reasonable medical costs. Because of this, many people perform brain MRI for health screening purposes even without suspecting neurological disorder. Therefore, the study population of this study can be interpreted as a general population without serious neurological history.

### Demographic, clinical, and laboratory findings

We evaluated various demographic and clinical factors including age, sex, body mass index, hypertension, diabetes, hyperlipidemia, ischemic heart disease, current smoking, use of antiplatelet agents, and systolic and diastolic blood pressure (BP) [15].

Laboratory evaluations were performed after overnight fasting or at least 12 h [15]. Laboratory evaluation included hemoglobin A1c (%), fasting glucose (mmol/L), total/low-density lipoprotein (LDL)/HDL cholesterol (mmol/L), triglycerides (mmol/L), white blood cell (WBC) counts (× 10^3^/μL), neutrophil/lymphocyte/monocyte counts (× 10^3^/μL), and high-sensitivity C-reactive protein (hs-CRP) (mg/dL). MHR was calculated by dividing the monocyte count by HDL cholesterol, as follows: MHR = monocyte counts (× 10^3^/μL) / HDL cholesterol (mmol/L). The NLR was calculated as the ratio of neutrophil and lymphocyte counts as follows: NLR = neutrophil counts (× 10^3^/μL) / lymphocyte counts (× 10^3^/μL) [16, 17, 22].

### Radiological findings

All participants underwent brain MRI and angiography (MRA) using 1.5-T MR scanners (Signa, GE Healthcare, Milwaukee, WI, USA or Magnetom, SONATA, Siemens, Munich, Germany) on the same day as the other tests. Detailed information about each MRI acquisition was as follows: basic slice thickness = 5 mm, T1-weighted images: repetition time (TR)/echo time (TE) = 500/11 ms, T2-weighted images: TR/TE = 5,000/127 ms, T2 fluid-attenuated inversion recovery images (FLAIR): TR/TE = 8,800/127 ms, T2-gradient echo images: TR/TE = 57/20 ms, and three-dimensional time-of-flight MRA images: TR/TE = 24/3.5 ms, slice thickness = 1.2 mm.

As subtypes of cSVD, we evaluated three pathologies: WMH, lacunes, and CMBs. The volume of WMH was quantitatively measured using Medical Imaging Processing, Analysis, and Visualization software (MIPAV, version, 11.0.0, National Institutes of Health, Bethesda, MD, USA). To accomplish this, each participant’s MRI image was obtained in the form of a DICOM file and then entered into the program for analysis. We specified the boundary line of the WMH lesions observed on T2 FLAIR images for each slice and combined them to calculate the volume in a semi-automated manner [15]. Lacunes were defined as well-defined asymptomatic lesions ranging from 3 to 15 mm in the territories of perforating arterioles with signal characteristics similar to those of cerebrospinal fluid on T1- or T2-weighted images [5]. CMBs were defined as focal round lesions less than 10 mm in size with low signal intensity on T2-gradient echo images [5]. For lacunes and CMBs, disease burden was measured as absent, single, or multiple, according to the number of lesions. All radiological parameters were rated by two neurologists (K.-W.N. and H.-Y.J.). Disagreements were resolved through discussion with a third rater (H.-M.K.).

### Statistical analysis

All statistical analyses were performed using SPSS version 23.0 (IBM Corp., Armonk, NY, USA). Continuous variables with normal distributions were presented as the mean ± standard deviation and the others were shown as the median [interquartile range]. Continuous variables with skewed data were transformed to log scales. Exceptionally, only the WMH volume was transformed into a squared-root scale because many participants had a value of zero.

To determine the characteristics of participants with high MHR, we compared the demographic, clinical, laboratory, and radiological findings according to MHR tertiles. The Kruskal–Wallis test, Jonckheere-Terpstra test, and chi-square test were used for this analysis.

To perform univariate analysis, we used simple linear regression analysis for WMH volume. For binary outcomes such as lacunes and CMBs, Student’s t-test, Mann–Whitney U-test, chi-squared test, and Fisher’s exact test were appropriately used according to the characteristics of the variables. Variables with *P* < 0.05 as a result of univariate analysis were introduced to multivariable linear or logistic regression analyses. Using NLR, another well-known subclinical inflammation marker, we performed sensitivity analyses using the same statistical methods.

We analyzed not only the association but also the quantitative relationship between MHR and cSVD subtypes. The MHR values according to the disease burdens of WMH, lacunes, and CMBs were compared, and the Jonckheere-Terpstra test was used for this analysis. All variables with *P* < 0.05 were considered significant in this study.

## Results

A total of 3,144 participants were evaluated (mean age: 56 ± 9 years, male sex: 53.9%). The mean MHR value was 0.28 ± 0.14 and the mean WMH volume was 2.64 ± 6.36 mL. The prevalence of lacunes and CMBs were 230 (7.3%) and 129 (4.1%), respectively. The other detailed baseline characteristics are presented in Table 1.

**Table 1:** Baseline characteristics of the cohort (*n* = 3,144)

	**Total**
Age, y [IQR]	56 [50–63]
Sex, male, n (%)	1,696 (53.9)
Body mass index, kg/m^2^ [IQR]	24.00 [22.11–25.93]
Hypertension, n (%)	791 (25.2)
Diabetes, n (%)	462 (14.7)
Hyperlipidemia, n (%)	480 (15.3)
Ischemic heart disease, n (%)	108 (3.4)
Current smoking, n (%)	565 (18.0)
Use of antiplatelet agents, n (%)	317 (10.1)
Systolic blood pressure, mmHg [IQR]	125 [115–136]
Diastolic blood pressure, mmHg [IQR]	75 [69–83]
Hemoglobin A1c, % [IQR]	5.7 [5.5–6.0]
Fasting glucose, mmol/L [IQR]	5.06 [4.72–5.61]
Total cholesterol, mmol/L [IQR]	5.12 [4.50–5.74]
LDL cholesterol, mmol/L [IQR]	3.23 [2.64–3.83]
HDL cholesterol, mmol/L [IQR]	1.37 [1.16–1.63]
Triglyceride, mmol/L [IQR]	1.13 [0.82–1.63]
White blood cell counts, × 10^3^/μL [IQR]	5.32 [4.40–6.38]
Neutrophil counts, × 10^3^/μL [IQR]	2.88 [2.22–3.68]
Lymphocyte counts, × 10^3^/μL [IQR]	1.86 [1.54–2.22]
Monocyte counts, × 10^3^/μL [IQR]	0.34 [0.27–0.44]
High-sensitivity CRP, mg/dL [IQR]	0.04 [0.01–0.15]
Neutrophil to lymphocyte ratio, [IQR]	1.53 [1.18–2.03]
Monocyte to HDL cholesterol ratio, [IQR]	0.25 [0.18–0.35]
WMH volume, mL [IQR]	1.04 [0.20–2.60]
Lacunes of presumed vascular origin, n (%)	230 (7.3)
Cerebral microbleeds, n (%)	129 (4.1)

*LDL* low-density lipoprotein, *HDL* high-density lipoprotein, *CRP* C-reactive protein, *WMH* white matter hyperintensity

**Fig. 1:**
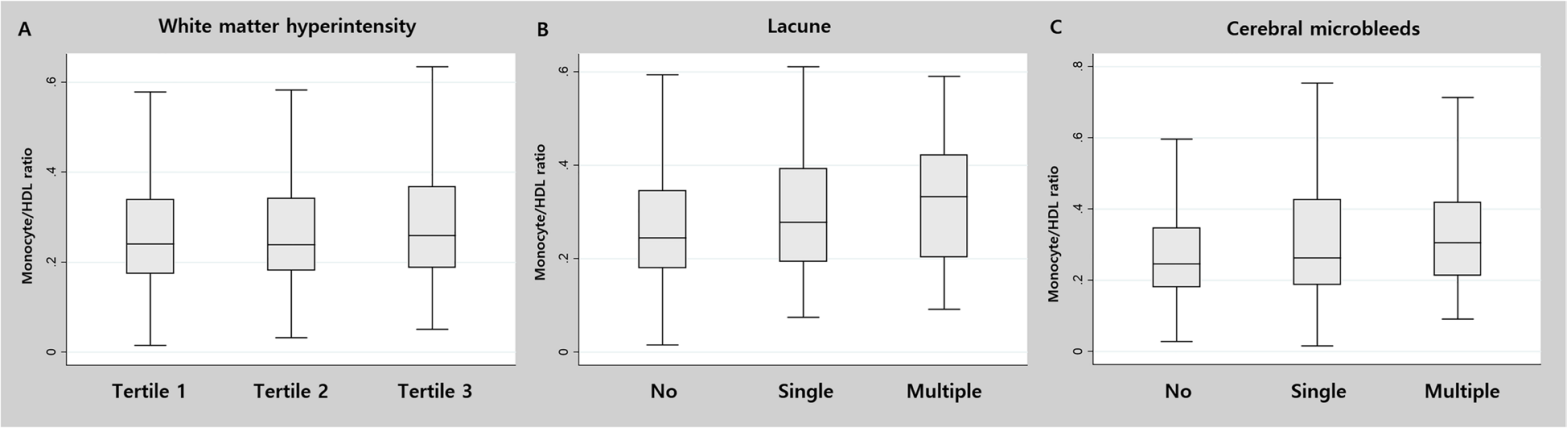
Quantitative relationships between monocyte to HDL cholesterol ratio and each subtype of cerebral small vessel disease. Monocyte to HDL cholesterol ratio showed a positive quantitative association with all types of cerebral small vessel disease, including white matter intensity volume (*P* < 0.001), lacunes (*P* < 0.001), and cerebral microbleed (*P* < 0.001)

In comparison between MHR tertiles, as MHR value increased, the frequency of male sex, hypertension, diabetes, and current smoking increased. Additionally, BP was high, and glucose and lipid profiles, cell counts, and inflammatory markers also increased overall. The burden of cSVD lesions also increased as the MHR value increased (Fig. 1). Detailed data can be found in Table 2.

**Table 2:** Comparisons of baseline characteristics among the MHR tertiles

	**MHR Tertile 1** (MHR < 0.21)	**MHR Tertile 2** (0.21 ≤ MHR < 0.31)	**MHR Tertile 3** (0.31 ≤ MHR)	***P*-value**	***P*-trend**
Number	1,048	1,049	1,047		
Age, y [IQR]	56 [51–62]	56 [50–63]	56 [49–64]	0.898	0.926
Sex, male, n (%)	346 (33.0)	555 (52.9)	795 (75.9)	< 0.001	< 0.001
Body mass index	23.19 [21.30–25.08]	24.02 [22.32–25.95]	24.69 [22.94–26.64]	< 0.001	< 0.001
Hypertension, n (%)	218 (20.8)	268 (25.5)	305 (29.1)	< 0.001	< 0.001
Diabetes, n (%)	108 (10.3)	137 (13.1)	217 (20.7)	< 0.001	< 0.001
Hyperlipidemia, n (%)	166 (15.8)	168 (16.0)	146 (13.9)	0.344	0.228
Ischemic heart disease, n (%)	27 (2.6)	38 (3.6)	43 (4.1)	0.145	0.054
Current smoking, n (%)	74 (7.1)	149 (14.2)	342 (32.7)	< 0.001	< 0.001
Systolic blood pressure, mmHg [IQR]	123 [113–134]	126 [116–136]	127 [117–138]	< 0.001	< 0.001
Diastolic blood pressure, mmHg [IQR]	74 [67–82]	75 [69–83]	76 [70–85]	< 0.001	< 0.001
Hemoglobin A1c, % [IQR]	5.7 [5.4–5.9]	5.7 [5.5–6.0]	5.8 [5.5–6.2]	< 0.001	< 0.001
Fasting glucose, mg/dL [IQR]	5.00 [4.67–5.50]	5.06 [4.67–5.61]	5.17 [4.78–5.83]	< 0.001	< 0.001
Total cholesterol, mmol/L [IQR]	5.22 [4.60–5.82]	5.12 [4.50–5.77]	5.02 [4.40–5.66]	< 0.001	< 0.001
LDL cholesterol, mmol/L [IQR]	3.15 [2.53–3.72]	3.31 [2.74–3.93]	3.23 [2.61–3.87]	< 0.001	0.018
Triglyceride, mmol/L [IQR]	0.89 [0.70–1.20]	1.12 [0.86–1.56]	1.48 [1.07–2.12]	< 0.001	< 0.001
WBC counts, × 10^3^/μL [IQR]	4.46 [3.80–5.24]	5.29 [4.53–6.12]	6.43 [5.50–7.72]	< 0.001	< 0.001
Neutrophil counts, × 10^3^/μL [IQR]	2.37 [1.89–2.92]	2.83 [2.23–3.47]	3.53 [2.85–4.44]	< 0.001	< 0.001
Lymphocyte counts, × 10^3^/μL [IQR]	1.65 [1.36–1.94]	1.87 [1.56–2.17]	2.09 [1.76–2.50]	< 0.001	< 0.001
High-sensitivity CRP, mg/dL [IQR]	0.01 [0.01–0.07]	0.04 [0.01–0.13]	0.12 [0.01–0.24]	< 0.001	< 0.001
NLR, [IQR]	1.43 [1.09–1.92]	1.49 [1.17–1.96]	1.68 [1.28–2.18]	< 0.001	< 0.001
WMH volume, mL [IQR]	0.93 [0.19–2.42]	1.07 [0.20–2.60]	1.20 [0.20–2.90]	0.019	0.005
Lacunes, n (%)	62 (5.9)	68 (6.5)	100 (9.6)	0.003	0.001
Cerebral microbleeds, n (%)	35 (3.3)	37 (3.5)	57 (5.4)	0.027	0.015

*MHR* monocyte to HDL cholesterol ratio, *LDL* low-density lipoprotein, *WBC* white blood cell, *CRP* C-reactive protein, *NLR* neutrophil to lymphocyte ratio, *WMH* white matter hyperintensity

WMH volume was associated with age, hypertension, diabetes, current smoking, use of antiplatelet agents, systolic and diastolic BP, hemoglobin A1c, fasting glucose, LDL cholesterol, WBC, neutrophil, monocyte counts, NLR, and MHR in univariate linear regression analyses. In the multivariable linear regression analysis, MHR was significantly associated with WMH volume after adjusting for confounders [*β* = 0.099, 95% confidence interval (CI) = 0.025 to 0.174]. Age (*β* = 0.050, 95% CI = 0.046 to 0.054), hypertension (*β* = 0.163, 95% CI = 0.077 to 0.249), diabetes (*β* = 0.143, 95% CI = 0.002 to 0.284), and systolic BP (*β* = 0.006, 95% CI = 0.002 to 0.010) were also associated with WMH volume, independent of MHR (Table 3).

**Table 3:** Univariate and multivariable analyses to evaluate the association between possible predictors and white matter hyperintensity volume

	**Univariable analysis**		**Multivariable analysis**	
	**B (95% CI)**	***P-*value**	**B (95% CI)**	***P-*value**
Age	0.053 (0.050 to 0.057)	< 0.001	0.050 (0.046 to 0.054)	< 0.001
Sex, male	0.025 (-0.054 to 0.103)	0.538	…	…
BMI	-0.001 (-0.013 to 0.012)	0.919	…	…
Hypertension	0.471 (0.382 to 0.559)	< 0.001	0.163 (0.077 to 0.249)	< 0.001
Diabetes	0.413 (0.304 to 0.523)	< 0.001	0.143 (0.002 to 0.284)	0.047
Hyperlipidemia	0.075 (-0.033 to 0.184)	0.174	…	…
IHD	0.113 (-0.101 to 0.327)	0.301	…	…
Current smoking	-0.194 (-0.296 to -0.093)	< 0.001	0.029 (-0.067 to 0.126)	0.554
Use of anti-PLT	0.291 (0.162 to 0.420)	< 0.001	-0.060 (-0.180 to 0.060)	0.325
Systolic BP	0.011 (0.009 to 0.014)	< 0.001	0.006 (0.002 to 0.010)	0.003
Diastolic BP	0.008 (0.004 to 0.011)	< 0.001	-0.001 (-0.007 to 0.005)	0.732
HbA1*c	1.200 (0.860 to 1.541)	< 0.001	-0.346 (-0.846 to 0.154)	0.175
Fasting glucose^a^	0.670 (0.470 to 0.871)	< 0.001	0.075 (-0.193 to 0.343)	0.582
Total cholesterol	-0.038 (-0.080 to 0.004)	0.076	…	…
LDL cholesterol	-0.049 (-0.097 to -0.002)	0.041	…	…
HDL cholesterol	-0.037 (-0.144 to 0.071)	0.507	…	…
Triglyceride^a^	0.056 (-0.022 to 0.134)	0.162	…	…
WBC counts	0.046 (0.023 to 0.069)	< 0.001	…	…
Neutrophil counts	0.072 (0.042 to 0.102)	< 0.001	…	…
Lymphocyte counts	-0.018 (-0.089 to 0.052)	0.609	…	…
Monocyte counts	0.556 (0.275 to 0.836)	< 0.001	…	…
hs-CRP^a^	0.016 (-0.010 to 0.042)	0.235	…	…
NLR^a^	0.244 (0.152 to 0.337)	< 0.001	…	…
MHR^a^	0.138 (0.059 to 0.217)	0.001	0.099 (0.025 to 0.174)	0.009

*BMI* body mass index, *IHD* ischemic heart disease, *anti-PLT* antiplatelet agent, *BP* blood pressure, *HbA1c* hemoglobin A1c, *LDL* low-density lipoprotein, *HDL* high-density lipoprotein, *WBC* white blood cell, *hs-CRP* high-sensitivity C-reactive protein, *NLR* neutrophil to lymphocyte ratio, *MHR* monocyte to HDL cholesterol ratio

^a^These variables were transformed into log scales

In multivariable logistic regression analyses performed according to the results of univariate analyses (Tables S1 and S2), MHR showed a significant association with lacunes [adjusted odds ratio (aOR) = 1.43, 95% CI = 1.07–1.91] and CMB (aOR = 1.51, 95% CI = 1.03–2.19). In addition, lacunes showed a statistically significant association with age (aOR = 1.09, 95% CI = 1.07–1.11) and diastolic BP (aOR = 1.02, 95% CI = 1.00–1.05), while CMB showed a close association with age (aOR = 1.06, 95% CI = 1.04–1.08) and systolic BP (aOR = 1.01, 95% CI = 1.00–1.03, Table 4).

**Table 4:** Multivariable logistic regression analyses to evaluate the association between possible predictors and lacune/cerebral microbleeds

	**Lacune**		**Cerebral microbleeds**	
	Adjusted OR [95% CI]	*P-*value	Adjusted OR [95% CI]	*P-*value
Age	1.09 [1.07–1.11]	< 0.001	1.06 [1.04–1.08]	< 0.001
Sex, male	…	…	…	…
Body mass index	…	…	…	…
Hypertension	1.32 [0.99–1.78]	0.063	1.47 [0.99–2.16]	0.054
Diabetes	1.11 [0.68–1.81]	0.671	0.99 [0.63–1.57]	0.974
Hyperlipidemia	…	…	…	…
Ischemic heart disease	…	…	…	…
Current smoking	…	…	…	…
Use of anti-PLT	…	…	1.18 [0.71–1.96]	0.531
Systolic BP	1.00 [0.99–1.02]	0.720	1.01 [1.00–1.03]	0.023
Diastolic BP	1.02 [1.00–1.05]	0.045	…	…
Hemoglobin A1c	0.60 [0.22–1.62]	0.314	…	…
Fasting glucose	4.25 [0.66–27.30]	0.128	…	…
Total cholesterol	…	…	…	…
LDL cholesterol	…	…	…	…
HDL cholesterol	…	…	…	…
Triglyceride	…	…	…	…
WBC counts	…	…	…	…
Neutrophil counts	…	…	…	…
Lymphocyte counts	…	…	…	…
Monocyte counts	…	…	…	…
High-sensitivity CRP	…	…	…	…
NLR	…	…	…	…
MHR	1.43 [1.07–1.91]	0.016	1.51 [1.03–2.19]	0.033

*Anti-PLT* antiplatelet agent, *BP* blood pressure, *LDL* low-density lipoprotein, *HDL* high-density lipoprotein, *WBC* white blood cell, *CRP* C-reactive protein, *NLR* neutrophil to lymphocyte ratio, *MHR* monocyte to HDL cholesterol ratio

In the sensitivity analysis using NLR instead of MHR, NLR significantly associated with WMH volume (*β* = 0.156, 95% CI = 0.073 to 0.238). However, NLR was not significantly associated with lacunes (aOR = 1.06, 95% CI = 0.76–1.47) and CMB (aOR = 0.89, 95% CI = 0.58–1.37, Table S3).

## Discussion

In this study, a high MHR was associated with cSVD lesions on MRI in health check-up participants. The MHR showed a close association with all subtypes of cSVD, even showing a positive quantitative relationship. Therefore, we demonstrated that inflammation is a common pathological mechanism that induces various cSVD pathologies. Furthermore, MHR performed better as a biomarker than NLR in indicating an association between cSVD and inflammation.

The exact pathological mechanisms that could explain the close association between MHR and cSVD remain unclear. We attempted to infer these mechanisms by considering the characteristics of monocytes and HDL cholesterol, which constitute the MHR. First, monocytes are closely associated with endothelial dysfunction. Monocytes penetrate the subendothelial space through the surface of activated or damaged endothelial cells, differentiate into foam cells, and secrete various inflammatory cytokines (e.g., TNF-a, IL-6, and IL-6) and chemokines [17, 18, 20, 22, 23, 26]. These substances induce focal and systemic inflammation, exacerbate endothelial dysfunction, and can even lead to impairment of the blood–brain-barrier (BBB) [5]. Impaired BBB increases permeability, which can cause periventricular infiltration of several toxic metabolites, resulting in damage to the surrounding nerve tissue [1, 6, 15]. In addition, the clearance of interstitial fluid through the glymphatic pathway can also be disrupted [1, 15]. These mechanisms are sufficient to create and exacerbate cSVD. Second, activated monocytes can induce microvascular and macrovascular atherosclerosis. It is widely known that macrophages and foam cells play key roles in atherogenesis [27]. Atherosclerosis of large vessels formed in this way can induce diffuse hypoperfusion, leading to the formation of WMH or lacunes [5, 28]. In addition, in a previous study conducted on patients with pontine infarction, MHR showed a close association with early neurological deterioration [29]. From these results, monocytes are thought to be also involved in micro-atherosclerosis in the perforating artery that directly induces cSVD. Third, circulating monocytes create a hypercoagulable state by secreting various substances including tissue factors [17, 18, 23, 24]. It can occlude perforating arteries by forming microthrombi, which may be involved in the development of WMH or lacunes [7]. Since this procoagulant effect can be suppressed by HDL cholesterol through p38 activation or phosphoinositide 3-kinase, high MHR may be more closely related to this mechanism [17]. Last, patients with a high MHR tend to be older and have multiple vascular risk factors. Previous studies have also shown that MHR is associated with various metabolic diseases, including hypertension, diabetes, and obesity, each of which is an independent risk factor for cSVD.

HDL cholesterol inhibits monocyte function via various pathways. HDL cholesterol inhibits monocyte progenitor cell proliferation and monocyte activation and prevents monocyte migration by inhibiting the expression of adhesion molecules in endothelial cells [17, 18, 23]. In addition, by transferring peripheral cholesterol to the liver, it inhibits monocyte uptake of oxidized LDL cholesterol [17, 23, 24, 26]. This prevents differentiation into foam cells and consequently prevents the inflammatory cascade from proceeding. In addition, regardless of monocyte, HDL cholesterol acts as an antioxidant by itself or induces NO secretion in endothelial cells to exert a neuroprotective effect [18, 25]. In conclusion, from the point of view of HDL cholesterol, it can be interpreted that high MHR does not prevent low HDL cholesterol from exercising the pathological influence of monocytes described above, and does not perform the function of protecting endothelial cells and nerve cells on its own, resulting in cSVD.

Interestingly, in our data, MHR showed close associations with all cSVD subtypes, whereas NLR only showed an association with WMH volume. We investigated why MHR showed a better association with cSVD, even though NLR is a better-known inflammatory marker than MHR. In previous studies, the NLR showed excellent predictive power for various prognoses in patients with acute ischemic stroke [30–32]. When a stroke event occurs, the sympathetic tone rises, and as a result, neutrophils and monocytes are released from the bone marrow into the circulating blood [33]. Meanwhile, as the spleen is suppressed, lymphopenia also occurs [33]. Since this phenomenon appears proportional to stroke burden, NLR could show a close association with various acute ischemic stroke outcomes [32]. However, cSVD is a chronic subclinical disease that does not produce acute stress events that are sufficient to induce lymphopenia. Conversely, monocytes are increased in various chronic stressful environments including obesity, diabetes, and hypertension [26, 34]. Low HDL cholesterol is also an indicator of the chronic metabolic status of lipids and an indicator of inhibition of monocyte function. By combining high monocyte count and low HDL cholesterol, MHR appears to be superior to NLR in reflecting the chronic effects of inflammation on cSVD pathologies.

MHR is an indicator that can be obtained easily, simply, and inexpensively through a simple blood test that is now performed in most health check-ups [17]. In addition, it has been recognized as a stable inflammatory marker and has recently been used for diagnosis and prognosis in various inflammatory, vascular, and metabolic diseases [35–40]. This is likely to be the case in the area of cerebrovascular disease as well, and our findings suggest the clinical possibility that high MHR values may be helpful in selecting patients at high risk for cSVD who require brain MRI. Korea has relatively good access to brain MRI, but this will also be helpful in terms of health care efficiency in countries with poor medical environments.

Interpreting our results entails several limitations. First, because this was a retrospective cross-sectional study, we can only suggest an association between MHR and cSVD, but cannot guarantee a causal relationship. Second, we analyzed the association with cSVD using a single MHR value measured on the day of the health check-up. As cSVD is a chronic subclinical pathology, it may develop over many years. Therefore, if we analyze the association between MHR values over several time points and the formation and progression of cSVD, clear relationship can be inferred. Third, we did not measure periventricular and subcortical WMH and deep and lobar CMBs separately. Periventricular and subcortical WMH are known to have somewhat different pathological etiologies, and this is also true for lobar and deep CMB [7]. If these pathologies were classified by location and the association between each pathology and MHR was identified, the influence of inflammation on these pathologies could be clarified more clearly. Fourth, cell counts can be affected by various underlying diseases or drugs. Therefore, we must also consider the impact of various comorbidities that we did not include in our analysis. Last, the study population was relatively young and had few vascular risk factors. Therefore, the influence of accompanying comorbidities may be somewhat underestimated.

## Conclusion

In conclusion, MHR is closely related to all cSVD subtypes. Since before, a lot of interest and research has been conducted on the possibility of anti-inflammatory treatment of cerebrovascular disease. We may be able to discover new treatments through research that monitors the effectiveness of anti-inflammatory treatment through multiple MHR measurements and tracks the improvement of cerebrovascular disease. However, these expectations must be verified in follow-up prospective studies.

### Supplementary Information

**Additional file 1: Table S1.** Differences of characteristics between patients with and without lacune. **Table S2.** Differences of characteristics between patients with and without cerebral microbleeds. **Table S3.** Sensitivity multivariable analyses between neutrophil to lymphocyte ratio and cerebral small vessel diseases.

## Data Availability

All data covered in this study are presented in the manuscript and the additional files.

## Abbreviations

cSVD: Cerebral small vessel disease
WMH: White matter hyperintensity
CMB: Cerebral microbleed
NLR: Neutrophil to lymphocyte ratio
HDL: High-density lipoprotein
MHR: Monocyte-to-HDL cholesterol ratio
BP: Blood pressure
LDL: Low-density lipoprotein
WBC: White blood cell
hs-CRP: High-sensitivity C-reactive protein
TR: Repetition time
TE: Echo time
FLAIR: Fluid-attenuated inversion recovery
