# Supplementary material for: Monocyte to high-density lipoprotein cholesterol ratio is associated with cerebral small vessel diseases
Source: BMC Neurol. 2024 Jan 4;24:18. doi: 10.1186/s12883-023-03524-9 (PMC10765827; doi:10.1186/s12883-023-03524-9)
Supplement: Supplementary file 1 — Additional file 1: Table S1. Differences of characteristics between patients with and without lacune. Table S2. Differences of characteristics between patients with and without cerebral microbleeds. Table S3. Sensitivity multivariable analyses between neutrophil to lymphocyte ratio and cerebral small vessel diseases. [file 12883_2023_3524_MOESM1_ESM.docx]

**Table S1. Differences of characteristics between patients with and without lacune**

|  | **No lacune**  **(n = 2,914)** | **Lacune**  **(n = 230)** | ***P*-value** |
| --- | --- | --- | --- |
| Age | 56 [50-62] | 63 [57-69] | < 0.001 |
| Sex, male | 1,566 (53.7) | 130 (56.5) | 0.415 |
| Body mass index | 24.00 [22.11-25.93] | 24.03 [22.03-26.09] | 0.746 |
| Hypertension | 699 (24.0) | 92 (40.0) | < 0.001 |
| Diabetes | 405 (13.9) | 57 (24.8) | < 0.001 |
| Hyperlipidemia | 449 (15.4) | 31 (13.5) | 0.433 |
| Ischemic heart disease | 98 (3.4) | 10 (4.3) | 0.430 |
| Current smoking | 528 (18.1) | 37 (16.1) | 0.440 |
| Use of anti-PLT | 286 (9.8) | 31 (13.5) | 0.076 |
| Systolic BP | 125 [115-136] | 130 [120-141] | < 0.001 |
| Diastolic BP | 75 [69-83] | 77 [70-85] | 0.002 |
| Hemoglobin A1c | 5.7 [5.5-6.0] | 5.9 [5.6-6.2] | < 0.001 |
| Fasting glucose | 5.06 [4.72-5.61] | 5.22 [4.72-6.11] | 0.006 |
| Total cholesterol | 5.12 [4.53-5.77] | 4.93 [4.34-5.64] | 0.009 |
| LDL cholesterol | 3.23 [2.64-3.83] | 2.97 [2.35-3.85] | 0.025 |
| HDL cholesterol | 1.37 [1.16-1.63] | 1.32 [1.11-1.58] | 0.044 |
| Triglyceride | 1.12 [0.82-1.62] | 1.23 [0.86-1.73] | 0.037 |
| WBC counts | 5.30 [4.40-6.37] | 5.51 [4.53-6.57] | 0.056 |
| Neutrophil counts | 2.86 [2.21-3.66] | 3.12 [2.32-3.96] | 0.014 |
| Lymphocyte counts | 1.86 [1.54-2.22] | 1.89 [1.53-2.20] | 0.947 |
| Monocyte counts | 0.34 [0.27-0.44] | 0.37 [0.28-0.47] | 0.006 |
| High-sensitivity CRP | 0.04 [0.01-0.15] | 0.07 [0.01-0.17] | 0.082 |
| NLR | 1.52 [1.17-2.01] | 1.66 [1.28-2.14] | 0.011 |
| MHR | 0.25 [0.18-0.35] | 0.28 [0.20-0.39] | 0.001 |

anti-PLT = antiplatelet agent, BP = blood pressure, LDL = low-density lipoprotein, HDL = high-density lipoprotein, WBC = white blood cell, CRP = C-reactive protein, NLR = neutrophil to lymphocyte ratio, MHR = monocyte to HDL cholesterol ratio

**Table S2. Differences of characteristics between patients with and without cerebral microbleeds**

|  | **No CMB**  **(n = 3,016)** | **CMB**  **(n = 128)** | ***P*-value** |
| --- | --- | --- | --- |
| Age | 56 [50-62] | 62 [56-68] | < 0.001 |
| Sex, male | 1,618 (53.7) | 78 (60.5) | 0.129 |
| Body mass index | 24.00 [22.10-25.94] | 24.07 [22.11-25.73] | 0.974 |
| Hypertension | 738 (24.5) | 53 (41.1) | < 0.001 |
| Diabetes | 435 (14.4) | 27 (20.9) | 0.041 |
| Hyperlipidemia | 461 (15.3) | 19 (14.7) | 0.862 |
| Ischemic heart disease | 103 (3.4) | 5 (3.9) | 0.779 |
| Current smoking | 550 (18.2) | 15 (11.6) | 0.055 |
| Use of anti-PLT | 296 (9.8) | 21 (16.3) | 0.017 |
| Systolic BP | 125 [115-136] | 129 [118-142] | 0.003 |
| Diastolic BP | 75 [69-83] | 76 [70-84] | 0.194 |
| Hemoglobin A1c | 5.7 [5.5-6.0] | 5.8 [5.5-6.2] | 0.185 |
| Fasting glucose | 5.06 [4.72-5.61] | 5.11 [4.78-5.78] | 0.287 |
| Total cholesterol | 5.12 [4.50-5.74] | 5.09 [4.53-5.72] | 0.996 |
| LDL cholesterol | 3.23 [2.61-3.83] | 3.18 [2.77-3.88] | 0.809 |
| HDL cholesterol | 1.37 [1.16-1.63] | 1.32 [1.11-1.53] | 0.061 |
| Triglyceride | 1.12 [0.82-1.63] | 1.18 [0.84-1.64] | 0.762 |
| WBC counts | 5.31 [4.40-6.37] | 5.65 [4.64-6.56] | 0.032 |
| Neutrophil counts | 2.87 [2.21-3.67] | 3.14 [2.30-3.97] | 0.063 |
| Lymphocyte counts | 1.86 [1.53-2.21] | 1.90 [1.63-2.36] | 0.095 |
| Monocyte counts | 0.34 [0.27-0.44] | 0.36 [0.28-0.48] | 0.037 |
| High-sensitivity CRP | 0.04 [0.01-0.15] | 0.05 [0.01-0.17] | 0.234 |
| NLR | 1.53 [1.18-2.03] | 1.53 [1.20-2.06] | 0.532 |
| MHR | 0.25 [0.18-0.35] | 0.27 [0.19-0.42] | 0.005 |

CMB = cerebral microbleed, anti-PLT = antiplatelet agent, BP = blood pressure, LDL = low-density lipoprotein, HDL = high-density lipoprotein, WBC = white blood cell, CRP = C-reactive protein, NLR = neutrophil to lymphocyte ratio, MHR = monocyte to HDL cholesterol ratio

**Table S3. Sensitivity multivariable analyses between neutrophil to lymphocyte ratio and cerebral small vessel diseases**

|  | Univariate analysis | | Multivariable analysis | |
| --- | --- | --- | --- | --- |
|  | B (95% CI) or adjusted OR [95% CI]^*^ | *P-*value | B (95% CI) or adjusted OR [95% CI]^*^ | *P-*value |
| White matter hyperintensity volume | 0.244 (0.152 to 0.337) | < 0.001 | 0.156 (0.073 to 0.238) | < 0.001 |
| Lacune | 1.35 [0.99-1.85] | 0.060 | 1.06 [0.76-1.47] | 0.747 |
| Cerebral microbleeds | 1.08 [0.71-1.65] | 0.707 | 0.89 [0.58-1.37] | 0.593 |

^*^B value or adjusted odds ratio value of neutrophil to lymphocyte ratio adjusted as a confounder for variables with *P* < 0.05 in the results of univariate analysis
